# Type III Interferons, IL-28 and IL-29, Are Increased in Chronic HCV Infection and Induce Myeloid Dendritic Cell-Mediated FoxP3+ Regulatory T Cells

**DOI:** 10.1371/journal.pone.0044915

**Published:** 2012-10-10

**Authors:** Angela Dolganiuc, Karen Kodys, Christopher Marshall, Banishree Saha, Shuye Zhang, Shashi Bala, Gyongyi Szabo

**Affiliations:** Department of Medicine, University of Massachusetts Medical School, Worcester, Massachusetts, United States of America; Beijing Institute of Infectious Diseases, China

## Abstract

**Background & Aims:**

Hepatitis C virus (HCV) is difficult to eradicate and type III interferons (IFN-λ, composed of IL-28A, IL-28B and IL-29) are novel therapeutic candidates. We hypothesized that IFN-λ have immunomodulatory effects in HCV- infected individuals.

**Materials and Methods:**

We analyzed the expression of IFN-λ and its receptor (composed of IL-10R2 and IFN-λR subunits) in the blood and livers of patients with chronic (c)HCV infection compared to controls (those who cleared HCV by sustained virological response, SVR, and those with liver inflammation of non-viral origin, non-alcoholic steatohepatitis, NASH). We also compared the proliferative capacity of dendritic cells (DCs) obtained from healthy individuals and those with chronic HCV using a mixed leukocyte reaction combined with 3H-Td incorporation. In addition, the composition of the IFN-λ receptor (IFN-λR) on myeloid DCs, plasmacytoid DCs, PBMCs, and T cells was determined by FACS analysis.

**Results:**

We report that the expression of IFN-λ protein in serum and mRNA in liver is increased in cHCV patients, but not in those with HCV SVR or NASH, compared to controls. Liver level of IFN-λR mirrored the expression of serum IFN-λ and was higher in cHCV, compared to controls and HCV-SVR patients, suggesting that elevation of IFN-λ and IFN-λR are HCV-dependent. We further identified that innate immune cell populations expressed complete IFN-λ receptor. *In vitro*, recombinant IFN-λ promoted differentiation of monocyte-derived dendritic cells (DCs) into a phenotype with low T cell stimulatory capacity and high PD-L1 expression, which further promoted expansion of existing regulatory T cells. IFN-λ-DCs failed to induce *de novo* generation of regulatory T cells. The inhibitory capacity of IFN-λ-DCs was counteracted by recombinant IL-12 and by neutralization of the PD-1/PD-L1 system.

**Conclusions:**

Our novel findings of the immunomodulatory effect of IFN-λ contribute to the understanding of the anti-inflammatory and/or anti-viral potential of IFN-λ in cHCV.

## Introduction

Chronic infection with Hepatitis C virus (cHCV) is present in 3% of the world’s population with prevalence ranging from 0.1–5% in different European countries [1]. HCV is currently treated with a combination of interferon alpha and ribavirin, however a sustained virological response (SVR) is achieved only in ∼50% of cases [1], [2]. More recently IFN-lambda (IFN-λ) has emerged as a potential new therapeutic option for HCV infection. Elevated IFN- λ transcripts were identified in the livers and in the peripheral blood mononuclear cells (PBMCs) of patients with cHCV [3], [4]. *In vitro* IFN- λ is a potent inhibitor of HCV replication [4], [5]. Preclinical and early clinical data indicated that IFN- λ was well tolerated in animals and presented minimal side effects. [6]. Further, genetic variations in IFN- λ genes may predict sustained virological response (SVR) to standard therapy [7].

**Table 1:** Clinical characteristics of study individuals.

Parameter	Value		
	Treatment-naïve	SVR	NASH
Age (years)	42±12	48±11	41±5
Male	20	17	6
Female	4	4	6
AST (U/l)	71±23	22±11	69±31
HCV viral load	1.92×10^6^±0.36×10^6^	0	0
Liver biopsies performed	22	16	12
Fibrosis	8	4	1
Stage 1–2	6	4	1
Stage 3–4	2	0	0
Liver inflammation	22	16	12
Score: 0–6	16	10	9
Score: 7–12	6	6	3

**Figure 1:**
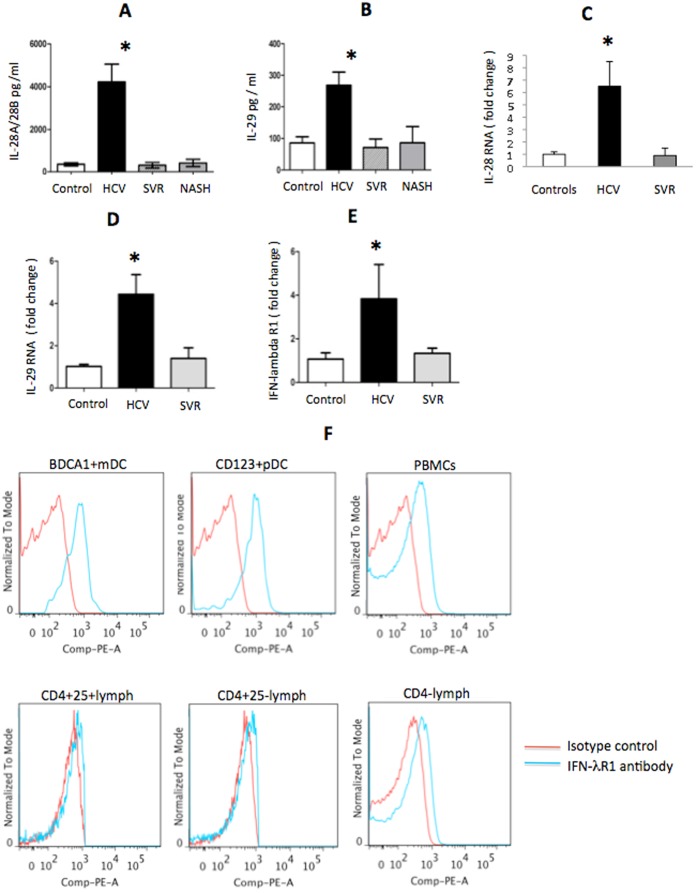
HCV infection correlates with high levels of IFN-λ and IFN-λR. Serum (A,B) and liver levels (C–E) of IL-28 (A-protein; C-mRNA), IL-29 (B-protein, D-mRNA), and IFN-λR (E, mRNA). There were 18 patients with chronic HCV infection, 12 controls, 16 HCV SVR and 12 NASH analyzed for serum cytokines (panels A,B). There were 18 patients with chronic HCV infection, 4 controls, and 16 SVR analyzed for RNA (panels C–E). The samples from blood (A,B) and liver RNA (C–E) were paired for HCV and SVR. (F) IFN-λR1 expression was analyzed in various cell types of PBMCs by flow cytometry using isotype or IFN-λR1 fluorescent antibody. Representative histogram of n = 4 is shown. * indicates p<0.05 compared to controls.

A total of 24 HCV, 21 SVR, 20 controls (serum and/or cells), 4 control liver RNA and 12 NASH serum were analyzed in our manuscript as follows:

• 18 HCV and 16 SVR pairs of blood and liver were analyzed for the data shown in Fig. 1.

• 12 control serum and 4 control liver mRNA were analyzed for the data shown in Fig. 1; these samples were not paired.

• 12 NASH blood (serum) were analyzed for the data shown in Fig. 1.

• The cells from the same 18 HCV analyzed in Fig. 1 were analyzed in Fig. 3 for their DC allostimulatory capacity.

• Additional 6 HCV, 5 SVR and 8 controls were recruited to perform the experiments shown in Fig. 3 in order to collect data for achieving sufficient statistical analysis power.

• Control liver RNA was of commercial origin from individuals without known liver diseases.

The IFN- λ class includes 3 cytokines, IL-29 (IFN- λ 1), IL-28A (IFN- λ 2), and IL-28B (IFN- λ3), which are produced upon stimulation with viruses or certain Toll-like receptor ligands mostly by hepatocytes, epithelial cells, and to a lesser extent by immune cells [5], [8], [9]. All IFN- λ class cytokines employ a common IFN- λ heterodimer receptor composed of a unique IFN- λ R1 chain and an IL-10R2 chain, the latter is also used by other cytokine receptors [8]. The signaling events downstream of IFN- λ R are shared with IFN-αR and include activation of STAT1, STAT2, and IRF9, all leading to induction of interferon-stimulated genes and antiviral activity. Taking into account that IFN- λ polymorphisms are associated with both treatment-induced and with natural HCV SVR [7], [10], it is likely that *in vivo* IFN- λ may be involved in anti-HCV innate immunity.

Innate immunity is key to antiviral defense. Innate immune defects have been identified in cHCV, including relative deficiency of circulating plasmacytoid dendritic cells (pDCs), altered expression of pathogen-recognition receptors, and a skewed monocytes/DC cytokine profile towards enriched production of immunoregulatory cytokines and impaired production of IFNs (reviewed in [11]), all leading to an inefficient T lymphocyte response and persistent cHCV. Regulatory T cells (Tregs) are specialized to suppress immune activation that are critical in the development of chronic viral infection and are phenotypically defined as naturally occurring, CD4+CD25+Foxp3+ or inducible Tregs. We and others have reported that cHCV is associated with an expanded Tregs population, both in peripheral blood and liver [12]–[14]. Treg functions in cHCV could be beneficial, by limiting inflammation and fibrosis (via direct contact with activated T cells and/or regulatory cytokines IL-10 and/or TGF-β, or detrimental, by creating a tolerant environment that favors tumor growth (reviewed in [15]). To date the mechanism of expansion and the role of Tregs in cHCV are not fully understood.

Here we report that IFN- λ levels in blood and liver are increased in cHCV. Further, we identified that IFN- λ enables generation of DC populations with regulatory capacity, which facilitates expansion of Foxp3+Tregs. These results suggest a novel role of IFN- λ in innate anti-HCV immunity.

## Materials and Methods

### Reagents

Recombinant cytokines IL-2, IL-4, GM-CSF and IFNs (IL-29 and IL-28A) were from Peprotech (Rocky Hill, NJ), anti-IL-10 antibodies (clone JES3-9D7) from Biosource, anti-PD-1 antibody from eBioscience (San Diego, CA), carboxyfluorescein-succinimidylester (CFSE) was from Invitrogen (Carlsbad, CA), and ^3^H-thymidine was from PerkinElmer (Waltham, MA).

### Blood Donors and Cell Culture

The study was approved by the Committee for Protection of Human Subjects in Research at University of Massachusetts Medical School and all individuals provided written consent to participate. Patients’ characteristics are described in Table 1. Core liver biopsies from patients were collected in our clinic and snap-frozen until analysis. Liver RNA from control individuals (free of liver disease) was purchased from Origene (n = 3) and from Stratagene (n = 1). Blood plasma was separated by centrifugation; PBMC were separated by centrifugation in Ficoll gradient; monocytes were isolated by adherence to plastic, as previously described [1]. Serum and cells were paired in controls; liver tissue was not paired with serum or cells in controls due to the commercial origin of normal liver RNA. When possible, paired serum/liver samples were analyzed in HCV and SVR patients.

**Figure 2:**
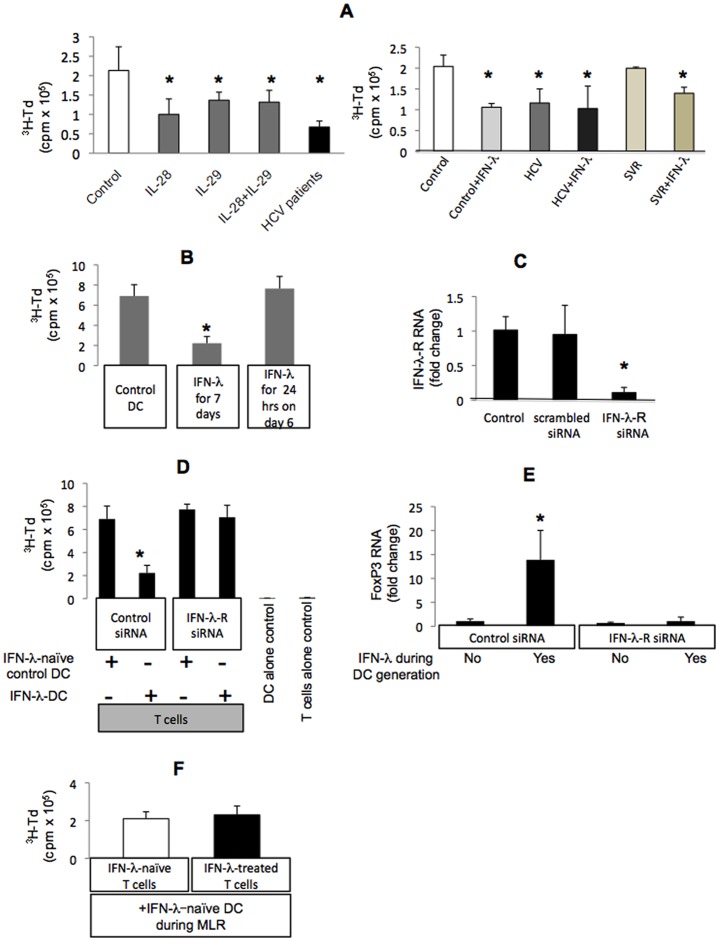
IFN-λ influences the DCs phenotype and functional capacity. Monocyte-derived DCs from healthy individuals were generated without (control) or with a combination of IL-29 and IL-28A (control+IFN-λ), or from patients with chronic HCV (HCV+IFN-λ) or recovered HCV infection (SVR+IFN-λ) for 7 days, after which they were included in a mixed lymphocyte reaction (MLR) with allogeneic CD4^+^ T cells for 4 days. Incorporation of ^3^H-Td during the last 16 hours of MLR, read as counts per minute (cpm), was considered indicative of the stimulatory capacity of DCs (A, left). DCs of controls, HCV and SVR individuals were analyzed as above (A, right). Control DCs, DCs generated for 7 days with IFN-λ, and DCs exposed to IFN-λ only during the last 24 hrs on day 6, were included in MLR. Incorporation of ^3^H-Td during the last 16 hours of MLR, read as cpm, was considered indicative of stimulatory capacity of DCs (B). Normal monocytes were transfected with scrambled or IFN-λR-specific siRNA and analyzed for the content of IFN-λR-coding RNA 72 hours later by RT-qPCR, as described in Methods. Fold change compared to mock-transfected cells was calculated from n = 3 sets of experiments (C). Normal monocytes were transfected as above, exposed to IL-4^+^GM-CSF in the presence or absence of IFN-λ for 7 days and included in MLR with normal T cells. T cells alone and DCs alone were used as controls of non-specific proliferation. Incorporation of ^3^H-Td during the last 16 hours of MLR, read as cpm, was measured as indicative of stimulatory capacity of DCs from n = 5 (D). Total RNA was extracted from MLR co-cultures corresponding to those described in panel D and the content of FoxP3-coding mRNA was analyzed using RT-qPCR, as described in methods. Fold change compared to scrambled MLR which used siRNA-exposed DCs as control was calculated from n = 5 sets of experiments (E). T cells of control healthy individuals were treated with IFN-λ for 5 days, after which they were washed extensively and co-cultured in MLR with dendritic cells which were generated with IL-4^+^GM-CSF without IFN-λ (IFN-λ-naïve) for 4 days. There was no IFN-λ present during MLR. Incorporation of ^3^H-Td during the last 16 hours of MLR, read as cpm, was measured as indicative of stimulatory capacity of DCs from n = 5 sets of experiments (F). * indicates p<0.05 compared to controls.

To test the effect on DC generation, the IFN λ (IL-29, IL-28A or their mixture) was added to adherent monocytes together with IL-4 and GM-CSF for 7 days. CD4^+^CD25^+^ (regulatory) T cells, CD4^+^CD25^−^ (effector) T cells, CD16^+^CD56^+^ NK cells, BDCA-1^+^ myeloid dendritic cells, CD123^+^BDCA-2^+^ plasmacytoid dendritic cells, and total CD4^+^ T cells were purified using magnetic beads (Miltenyi Biotech and StemCell Technologies), following the manufacturer’s instructions.

### Dendritic Cells and T Cells Function Assay

The T cell-stimulatory capacity of DCs was tested based on the principles of mixed lymphocyte reaction (MLR) as previously described [12] with slight modifications. Briefly, DCs were incubated with total CD4^+^ T cells in 96-well culture plates. On day four, 50% of culture supernatant was removed and subjected to cytokine quantification; the ^3^H-Td incorporation was evaluated using a beta-counter at the end of the final 16 h of the 5-day MLR and T cell proliferation was expressed as counts per minute (cpm). Alternatively, T cells were labeled with CFSE (10 µM), included into co-culture assay, recovered on day 10 and subjected to flow cytometry analysis or RNA extraction. For quantification of proliferation, CD4^+^ T cells were recovered from dendritic cell/Tcells co-culture, stained as detailed in the legends and analyzed for CFSE fluorescence by flow cytometry. For FoxP3 detection the cells were permeabelized using PermWash kit (BD Bioscience) prior to staining. After incubation with the antibodies the cells were washed, fixed with 1% paraformaldehyde in saline and analyzed by flow cytometry. Cells were gated based on their size and granularity and analyzed for fluorescence; data were expressed as histograms or dot blots.

**Table 2:** Phenotypic analyses of DCs generated in the presence or absence of IFN-λ.

Marker	Control DCs	IFN-λ-exposed DCs
CD80	MFI 465±212	488±156
CD86	MFI 214±46	209±84
CD14	MFI 126±101	131±66

To identify the inhibitory capacity of dendritic cell-expanded Tregs, the T cells were included into 2 rounds of MLR. The first MLR was set up as described above with CD4^+^ T cells as responders and control or IFN- λ -exposed DCs as stimulators. At the end of this first MLR, the T cells were recovered, washed, purified into CD4^+^CD25^−^ and CD4^+^CD25^+^ populations using magnetic beads, mixed at the indicated ratios and included into a second round MLR with allogeneic normal dendritic cells. Proliferation during the second MLR was assessed by incorporation of the ^3^H-Td incorporation during final 16 h of the 5-day co-culture.

*Cytokines* were quantified using specific ELISA kits, following the manufacturer’s instructions. IL-2, IL-12, and IL-10 kits were from BD Bioscience, IFN- λ 1 (IL-29) and IFN- λ 2 (IL-28A, cross-reacting with IL-28B) were from R&D Systems.

**Figure 3:**
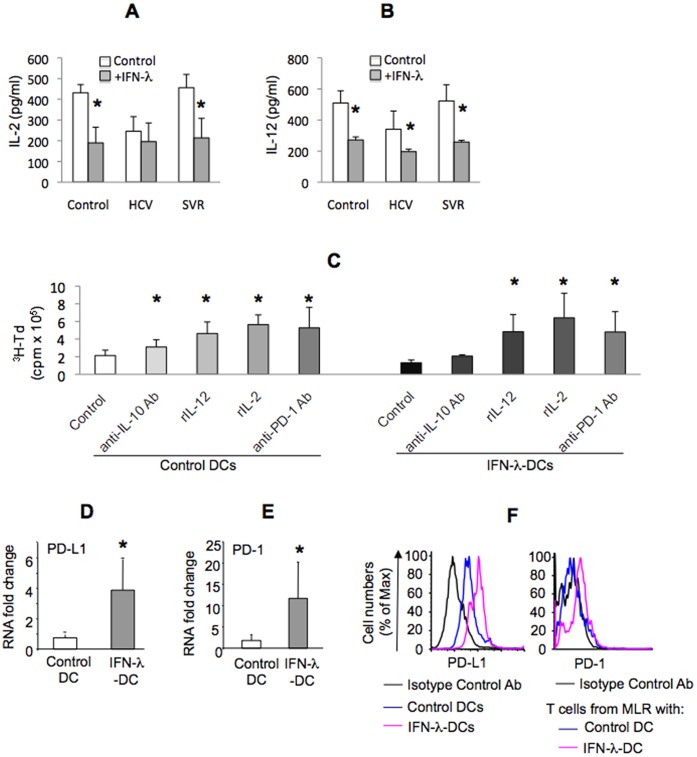
IFN-λ treatment-induced inhibitory DCs phenotype is dependent on IL-10 and PD-1/PD-L1 ligand. Monocyte-derived DCs from healthy individuals were generated without (control) or with IFN-λ (combination of IL-29 and IL-28A, called IFN-λ-DC) for 7 days, after which they were included in the mixed lymphocyte reaction (MLR) with allogeneic CD4^+^ T cells for 4 days. Co-culture supernatants from indicated groups were analyzed for IL-2 (A), IL-12 (B) by ELISA; mean±SD pg/ml shown. Neutralizing α-IL-10 or α-PD-1 antibodies, or recombinant (r) IL-2 or IL-12 were added during MLR and T cell proliferation was analyzed as described in Methods; shown mean±SD cpm from n = 5 sets of experiments (C). Entire MLR co-culture was analyzed for PD-L1(D) or PD-1 (E) mRNA. DCs were analyzed for PD-L1 expression by flow cytometry using isotype or specific fluorescent antibodies (MFI control 414±126 vs 622±98 IFN- λ DCs) (F, left). T cells from MLR co-culture cells were analyzed for PD-1 expression by flow cytometry using isotype or specific fluorescent antibodies (MFI control 125±27 vs 187±39 IFN- λ DCs) (F, right). * indicates p<0.05 compared to controls.

### RNA Analysis

Tissue or cell RNA was isolated with RNeasy Kit (Qiagen) and transcribed to cDNA with FirstStrand cDNA Synthesis Kit (Promega). Specific primers (all from IDT except 18S (Ambion)) and dsDNA-binding SYBR Green were used to quantify the gene products using iCycler software and comparative ΔCt method, as previously described [1]. The primers sequences were designed using http://frodo.wi.mit.edu/primer3/tool based on sequences identified in NCBI nucleotides database; primer sequences are shown in Table S1. The amplification efficiencies of the targets and the reference samples were within close range. The quantification of PCR data was achieved using the comparative C_t_ method. We calculated the 2^–ΔΔCt^, where ΔΔC_t_ = ΔC_t sample (patient or experimental group)_ − ΔC_t reference (control)._ The ΔC_T,sample_ was calculated as C_t_ value for any sample normalized to the endogenous housekeeping gene and ΔC_t, reference_ was the C_t_ value for the calibrator (normal control) also normalized to the endogenous housekeeping gene. The mean value of 2^–ΔΔCt^ from control group was considered equal to 1; the fold change over the mean 2^–ΔΔCt^ of controls was calculated for all samples by division. Data were expressed as mean+/− SD of fold change in every experimental group compared to control; this method of analysis is widely accepted in current literature.

**Figure 4:**
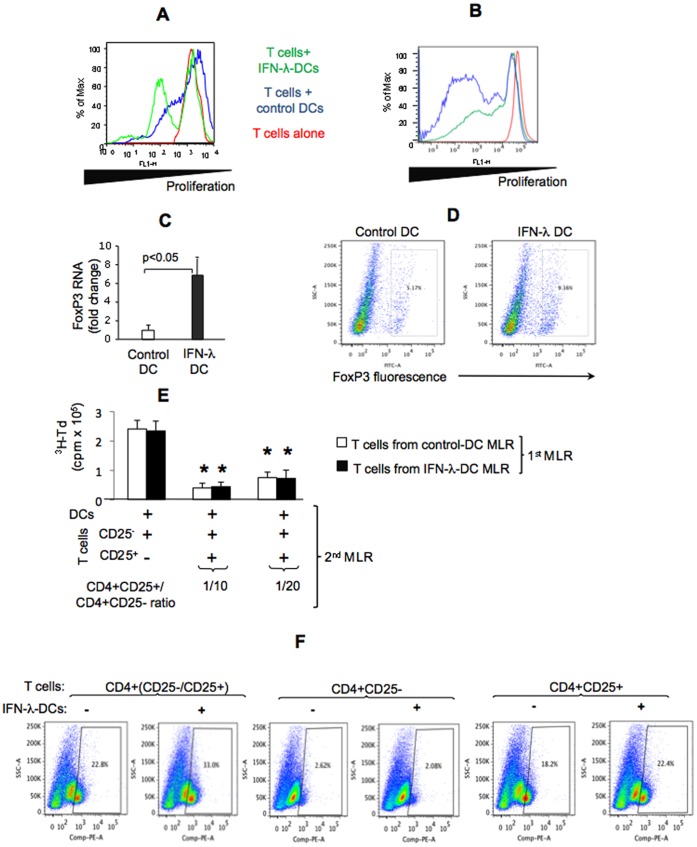
IFN-λ-DCs promote proliferation of existing but not *de novo* generation of regulatory T cells. (A,B) CD4^+^ T cells were labeled with CFSE and cultured alone (red) or co-cultured with control (blue) or IFN-λ-DCs (green); at the end of culture CD4^+^CD25^+^ cells (A) or CD4^+^CD25^−^ cells (B) were analyzed for CFSE content by flow cytometry; representative histogram of n = 8 is shown. (C,D) The DC/T cells co-culture was analyzed for FoxP3 mRNA (C) using specific primers in quantitative PCR or for the frequency of FoxP3^+^ cells by flow cytometry (D); data are shown as fold change compared to control DC/T cells co-culture (C) or as representative flow cytometry dot blots (D). (E) T cells were included into 2 rounds of MLR. The first MLR was set up as described in Fig. 3 with CD4^+^ T cells as responders and control or IFN- λ-exposed DCs as stimulators. At the end of this first MLR, the T cells were purified into CD4^+^CD25^−^ and CD4^+^CD25^+^ populations using magnetic beads and equal numbers of cell types were included into a second round MLR with allogeneic normal DCs at indicated DC/T cell ratio. Proliferation during the second MLR was assessed by ^3^H-Td incorporation during the final 16 h of the 5-day co-culture. Mean±SD cpm values from n = 8 are shown. (F) IFN- λ-exposed DCs were co-cultured with CD4^+^ (including CD25^+^ and CD25^-^ populations) or CD4^+^CD25^−^ or CD4^+^CD25^+^ T cells; T cell cultures without DCs were used as controls. The co-cultures were analyzed by flow cytometry for the frequency of FoxP3^+^ cells (F). Representative set of flow cytometry dotblots of n = 4 with similar results is shown. * indicates p<0.05 compared to controls.

The liver RNA of controls (n = 4) was purchased from Origene and Stratagene; according to the provider the donors were healthy and did not have liver diseases. This source of normal liver RNA was used previously by us [16] and others (European Patent EP1494031) due to worldwide restricted access to normal liver tissue. Further, we did not use peri-tumoral liver tissue as a control due to potential implications of IFN- λ in tumorigenesis, as recently described by Yang L et al [17]. We had four samples of normal liver; these samples were very close in their levels of all analyzed markers (Fig. 1). Further, these normal liver samples were run side-by-side with patient samples every time a PCR analysis was performed to provide a reliable inter-experimental control.

### Fluorescent Antibodies for FACS Analyses

Anti-human Lineage-1 FITC, anti-human HLA-DR PerCp, anti-human BDCA1 APC, anti-human CD123 PE-Cy7, anti-human CD56 APC, anti-human CD3 PerCp, anti-human CD4 PE-Cy7, anti-human CD25 APC-Cy7 and anti-human IL-28Ra were purchased from Biolegend (USA). Anti-human IL-10Rb PE was purchased from Abcam (UK).

### Statistical Analysis

The Wilcoxon analysis in the Statview (SAS Institute) program was employed for cytokines and mRNA quantification. For flow cytometry analysis, the statistical parameters from the FlowJo and CellQuest programs were employed. P values <0.05 were considered significant.

## Results

### IFN- λ and IFN- λ R Levels are Increased in Patients with Chronic HCV Infection, but not in those who Achieved SVR or in Liver Inflammation of Non-viral Etiology

In order to dissect the role of cHCV-induced inflammation in type III IFN production, we examined IFN-λ levels from the blood and livers of cHCV patients and control patients (Table 1). Serum IL-28A (Fig. 1A) and IL-29 (Fig. 1B) levels were elevated in patients with cHCV compared to controls. We also identified elevated liver mRNA levels of IL-28 (Fig. 1C) and IL-29 (Fig. 1D) in patients with cHCV compared to controls, which mirrored the increased serum levels of IFN-λ (Fig. 1A,B). cHCV is associated with chronic inflammation in the liver, which could be due to both the virus and immune-mediated reaction to the virus. We thus recruited 2 additional cohorts of patients: those who achieved sustained viral response (SVR) after treatment and those with non-alcoholic steatohepatitis (NASH); the former exhibited liver inflammation of non-viral origin (Table 1). Serum protein (Fig. 1A,B) and liver RNA (Fig. 1C,D) IFN- λ levels of SVR and NASH patients were comparable to controls and significantly lower compared to the cHCV cohort. Liver IFN- λR mRNA mirrored the serum IFN- λ levels and was elevated in the cHCV group compared to control and SVR groups (Fig. 1E). These data suggested that viral presence was needed to trigger/maintain the elevated IFN- λ levels during cHCV.

### IFN- λR is Expressed Differentially among Immune Cells

Close interactions between immune cells and HCV-infected hepatocytes occur in the liver. Having identified that IFN- λ and IFN- λR are elevated in the livers of cHCV patients (Fig. 1A–E), we sought to determine the composition of IFN- λR on immune cells. FACS analysis revealed significant expression of IFN- λR on BDCA-1^+^ myeloid dendritic cells (DC), CD123^+^BDCA-2^+^ plasmacytoid DCs, and PBMCs and to a lesser extent on CD4^+^CD25^+^ (regulatory) T cells, CD4^+^CD25^−^ (effector) T cells, and non-CD4^+^ T cells (Fig. 1F). The IFN- λ receptor has 2 components: a unique IFN- λR1 chain and an IL-10R2 which is shared with other receptors [8]. FACS analyses revealed IL-10R2 expression in the individual immune cell populations (data not shown). At the RNA level, both IFN- λR1 chain and IL-10R2 components of IFN- λR were characteristic for innate immune cells, including DCs (Fig. S1), which we subsequently targeted for further investigation. We also noted similar levels of expression of many of the analyzed markers in the analyzed cell types when compared between controls and HCV patients (Fig. S2A), except for higher FoxP3 and lower BDCA-2 levels in PBMCs in cHCV patients compared to controls (Fig. S2B); this observation was in agreement with previous reports of increased frequency of Tregs and decreased numbers of pDCs in blood of HCV patients [12], [15], [18].

### IFN-λ Facilitates the Differentiation of DCs with Inhibitory Capacity

Dendritic cells play a central role in the innate immune response. Here we report that *in vitro* treatment of monocytes with IL-28 or IL-29 did not interfere with the capacity of IL-4+GM-CSF to induce their differentiation into DCs, based on similar high CD80, CD86 and low CD14 levels (Table 2). In contrast, the presence of IL-28A, IL-29, or their combination, lead to development of DCs with decreased T cell stimulatory capacity (Fig. 2A left). To this end, IFN- λ-exposed DCs of healthy individuals were similar to primary DCs of cHCV patients (Fig. 2A right). We further identified that IFN- λ treatment was inhibitory for DCs of SVR individuals similar to healthy individuals (Fig. 2A right). Importantly, IFN- λ did not further suppress or restore the already impaired stimulatory capacity of cHCV-DCs (Fig. 2A right). The presence of IFN- λ for the entire duration of DC generation was necessary to establish the development of inhibitory DCs; limited exposure to IFN- λ at the end of DC generation did not affect the stimulatory properties of DCs (Fig. 2B). Exposure of monocytes to anti-IFN- λR siRNA, but not to non-specific scrambled siRNA, caused down-regulation of IFN- λR expression (Fig. 2C). More importantly, anti-IFN- λR siRNA-treated monocytes which had differentiated to DCs in the presence of IFN- λ were protected from the IFN- λ treatment-induced inhibitory phenotype of DCs, as indicated directly by lack of functional inhibitory activity of these DCs (Fig. 2D) and indirectly by the corresponding lack of FoxP3 increase in mixed lymphocyte reaction (MLR) (Fig. 2E). These data suggested that the effect of IFN- λ treatment on DCs is IFN- λR-dependent. In contrast, pre-exposure of T cells to IFN- λ prior to their contact with DCs did not affect their proliferation if IFN- λ-naïve control DCs were used as stimulators in IFN- λ-free co-culture conditions (Fig. 2F). Collectively, these data suggested that treatment with IFN- λ is inhibitory for DC function via IFN- λR on DCs.

### IFN-λ-exposed DCs Exhibit PD-1/PD-L1-dependent Regulatory Capacity

The stimulatory capacity of DCs is highly dependent on their cytokine and surface molecule profiles. HCV DCs triggered lower levels of IL-2 (Fig. 3A) and IL-12 (Fig. 3B) compared to control and SVR DCs in MLR. IL-2 (Fig. 3A) and IL-12 (Fig. 3B) levels were also decreased during MLRs with IFN- λ-exposed DCs when DCs originated from controls or SVR. Exposures of HCV DCs to IFN- λ lead to further inhibition of IL-12 but not IL-2 production (Fig. 3A&B). Neutralization of inhibitory factors (PD-1), or supplementation with stimulatory factors (IL-12 or IL-2), restored the stimulatory capacity of IFN-λ treated DC (Fig. 3C); neutralization of IL-10 had only minimal effects. We further identified increased levels of PD-L1 (Fig. 3D,F left) and PD-1 (Fig. 3E,F right) RNA (Fig. 3D,E) and protein (Fig. 3F) in MLRs with IFN-λ-treated DCs. These data suggested that IFN- λ-treated DCs created a regulatory environment that impairs T cell activation.

### IFN- λ-treated DCs Promote Proliferation of Regulatory T Cells

An environment with decreased IL-12 levels and increased expression of negative co-stimulatory molecules, such as PD-1/PD-L1 system, often leads to skewed composition of proliferating T cells. We found preferential proliferation of CD4^+^CD25^+^ cells in IFN- λ-DC/T cells, indicated by higher frequency of CFSE^low^ cells, compared to control DC/T cells co-cultures (Fig. 4A). The proliferation of CD4^+^CD25^−^ exposed to IFN- λ DCs was reduced, suggested by lower frequency of CFSE^low^ cells compared to control DCs in a MLR (Fig. 4B). We further identified increased expression of FoxP3 RNA levels (Fig. 4C) and higher frequency of FoxP3^+^ Tregs (Fig. 4D) in IFN- λ-DC/T cells compared to control DC/T cells co-cultures. These data suggested that IFN- λ-DCs favor proliferation of FoxP3^+^ T cells.

Tregs are very efficient in suppressing effector T cells. When present in comparable numbers, IFN- λ-DC-expanded Tregs have a comparable inhibitory capacity to Tregs previously exposed to control DCs (Fig. 4E). Further, IFN- λ-DC-expanded Tregs produce IL-10 and express CD45RA but not in excessive amounts compared to control cells (Fig. S3). These data suggested that the inhibitory effects of IFN- λ-DCs are likely due to their capacity to increase the numbers of Tregs via DC-dependent proliferation; their ability to induce Tregs with more powerful regulatory functions is unlikely.

### IFN- λ-DCs Promote Expansion of Pre-existing but not de novo Generation of Tregs

Finally, we addressed the origin of proliferating Tregs upon co-culture with IFN- λ-DCs. The frequency of FoxP3^+^ regulatory T cells (Fig. 4F) was increased upon T cell co-culture with IFN- λ-DCs when T cell population contained both CD4^+^CD25^+^ and CD4^+^CD25^−^ T cells. In contrast, no additional FoxP3-bearing cells were generated upon co-culture of IFN- λ-DCs with CD4^+^CD25^−^ lymphocytes (Fig. 4F). These results suggested that IFN- λ-DCs promote expansion of pre-existing, but not *de novo* generation of FoxP3^+^ T cells.

## Discussion

Although type III IFNs are currently in clinical trials as potential therapeutic agents to eradicate HCV infection, their mechanisms of action are not fully understood. Here we report that IFN- λ expression is increased in cHCV patients, both in blood and liver. Our results provide novel evidence for the immunomodulatory action of IFN- λ in anti-HCV immunity and add new complexity to the understanding of the anti-HCV potential of IFN- λ.

We found elevated levels of serum IFN- λ upon cHCV; these data are in agreement with some [19] but not other [20] recent studies. We further provide novel data that, in contrast to patients with active HCV infection, serum levels of IFN- λ in individuals with HCV SVR were comparable to HCV-naïve control. These data indicate that HCV presence is needed for increased IFN- λ production; similar findings were reported in other hepatotropic viral infections including HBV and and HIV [21], [22] infections. This was further supported by our observation, also confirmed by Diegelmann et al [19], that the presence of liver inflammation due to metabolic disturbances (such as in non-alcoholic steatohepatitis) did not lead to increased serum levels of IFN- λ.

We also found robust expression of the IFN- λ receptor (IFN- λR) in dendritic cells and to a lesser extent in T cell populations. We observed expression of the other component of the IFN- λR, IL-10R2, which is also shared between IL-10 and IL-22 [8]. While increased levels of IL-10 have been reported in HCV infection, to date there is no evidence for IL-10 modulating IFN- λR function. Our finding of the inhibitory effect of IFN-λ on dendritic cells indicates that distribution of IFN- λR may influence tissue-specific effects of IFN- λ during HCV infection.

HCV is difficult to eradicate and this could be related to its prolific capacity to manipulate the immune system. Here we showed that IFN- λ acts as a modulator of immune responses during HCV infection in three inter-related areas described below.

First, we demonstrated that IFN- λ directs DCs to a regulatory phenotype with increased expression of negative regulatory molecules. The IFN- λ-exposed DCs exhibited diminished capacity to stimulate T cell proliferation in a PD-1/PD-L1 dependent manner with contribution from the imbalanced cytokine milieu, such as low IL-12 and IL-2 and/or high IL-10 production. In fact, the functional potential of the IFN- λ-generated DCs, shown here, closely resembles the functional deficits seen in myeloid dendritic cells of HCV patients, as previously reported by our group, and others, although these changes were not identified in other patient cohorts (reviewed in [11], [15]). A limitation of our study is that monocyte-derived DCs may not fully represent the *in vivo* DC life cycle. However, our data suggest a PD-1/PD-L1-dependent effect of IFN- λ on the stimulatory capacity of DCs. We observed comparable effects of a PD-1 antibody on control DCs that express less PD-L1 levels thus lending support to a combinatory defect, including, but most likely not limited to, negative co-stimulatory receptors and cytokines. Nevertheless, the newly-discovered inhibitory effects of IFN-λ on DCs in our study are not surprising, taking into account the functional redundancy between IFN- λ and IFN-α and impaired DC response to IFN-α in patients with HCV infection [23].

Second, we show that IFN- λ-exposed DCs are potent triggers involved in the proliferation of Tregs. We and others reported increased frequency of Tregs upon HCV infection [12]–[14], [18]. Here we observed that IFN- λ-treated DCs trigger the proliferation of pre-existing Tregs and failed to induce *de novo* Treg generation.

Third, we found that the Tregs expanded in the presence of IFN- λ-DC have preserved their inhibitory capacity in a new MLR. These characteristics of the IFN- λ-DC-expanded Tregs closely resemble findings in primary CD4+CD25+FoxP3+ Tregs of HCV patients [12]–[15], [18], [24].

Finally, in light of the fact that IFN*-*λs induce a subset of ISGs stimulated by Type I IFNs and not a unique subset of genes [25], our novel finding of the immunomodulatory effects of IFN-λ on DCs require further investigations. Further studies will be needed to identify whether the immunomodulatory effect of IFN-λ on the MDCs/Tregs system may constitute a desirable anti-inflammatory potential, in parallel or in addition to its previously-reported direct anti-viral effects of IFN-λ against HCV [4], [5]. Alternatively, IFN-λ may play have a detrimental role due, at least in part, to its role in Tregs expansion.

In conclusion, we found increased levels of IFN-λ, IL-28, and IL-29 in serum and increased expression of these cytokines and their receptor in the liver during chronic HCV infection. We also identified the functional effect of IFN- λ on DCs, which results in dendritic cell-dependent expansion of regulatory T cells. Our study suggests that the immunomodulatory effects of IFN- λ on DC and T cell populations will need to be considered in future clinical studies involving IFN- λ therapy in HCV infection.

## Supporting Information

Figure S1 **Differential expression of IFN- λR in various cell types of PBMCs.** PBMCs or indicated immune cell populations, purified as described in Methods, were analyzed for listed genes using qPCR and specific primers; the amplified products were separated in agarose gel and representative blots from one representative individual are shown.
(TIF)

Figure S2 **cHCV patients have increased levels of FoxP3 and reduced BDCA-2 in PBMCs compared to controls.** Cell populations were isolated based on specific markers, as described in methods. Equal amounts of total cellular RNA was transcribed to DNA and analyzed for expression of specific markers by qPCR using SYBRgreen and specific primers. (A) PCR cycles, adjusted to housekeeping 18S control, are shown as mean ±SD. (B) The mean RNA levels of specific markers (FoxP3-top, BDCA-2-bottom) in PBMCs of controls were considered as equal to 1 and the fold change in HCV patient groups compared to controls was calculated (shown as mean±SD fold). * indicates p<0.05.
(TIF)

Figure S3 **Tregs expanded by IFN- λ-exposed DCs have a regulatory phenotype.** T cells were co-cultured with either control or IFN- λ-exposed DCs for 10 days, after which they were stimulated with PMA+Ionomycin for 4 hrs in the presence of GolgiStop. Cells were permeabilized, stained with specific antibodies, fixed, and analyzed by flow cytometry. Tregs were identified as CD4^+^CD25^+^FoxP3^+^ and analyzed for expression of CD45RA and IL-10 as indicated. Representative histograms of n = 5 are shown.
(TIF)

Table S1 Primers for PCR analyses.
(DOC)
