# Supplementary figures and images for: Type III Interferons, IL-28 and IL-29, Are Increased in Chronic HCV Infection and Induce Myeloid Dendritic Cell-Mediated FoxP3+ Regulatory T Cells
Source: PLoS One. 2012 Oct 10;7(10):e44915. doi: 10.1371/journal.pone.0044915 (PMC3468613; doi:10.1371/journal.pone.0044915)

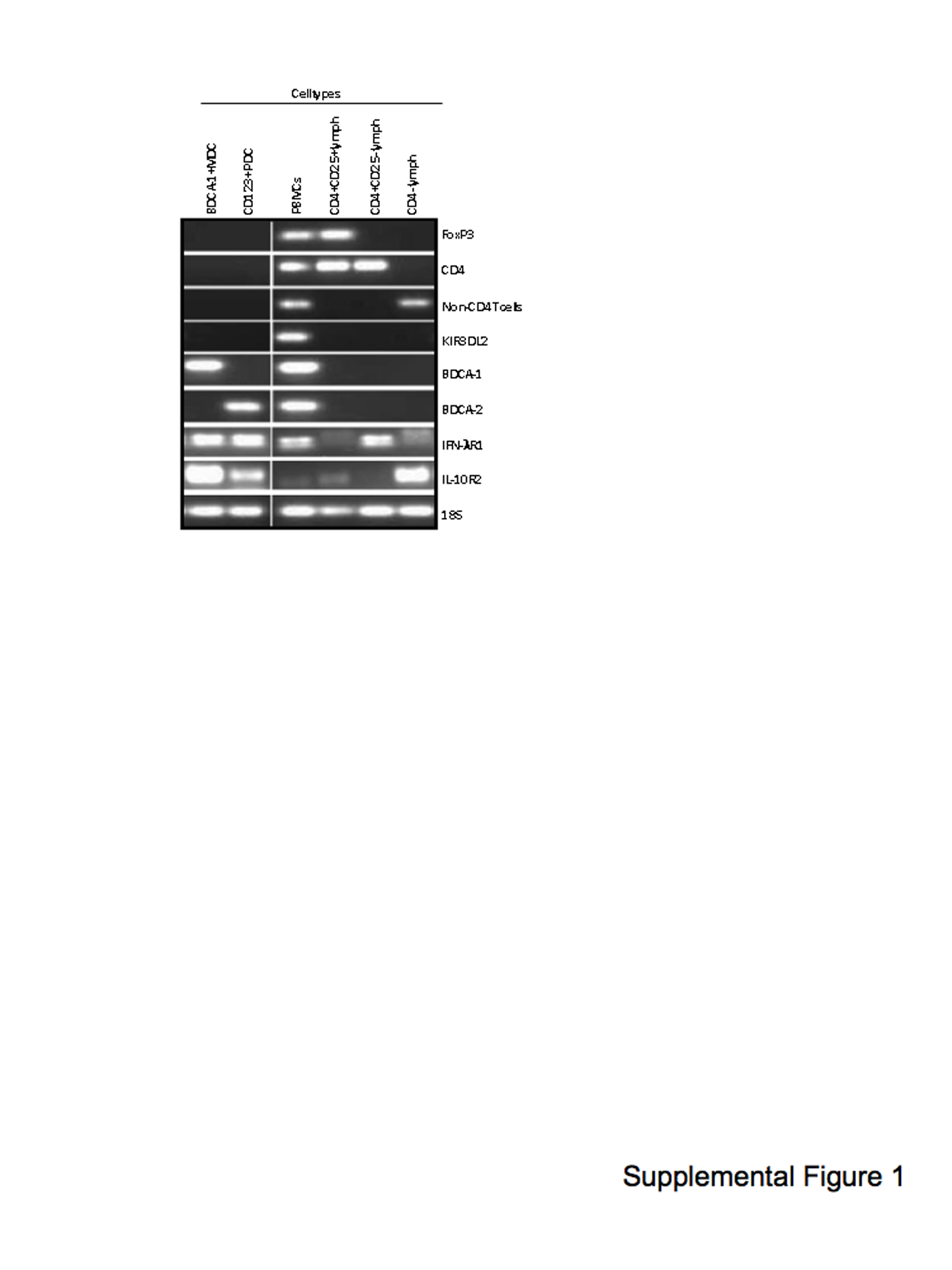

Supplement: Figure S1 — Differential expression of IFN- λR in various cell types of PBMCs. PBMCs or indicated immune cell populations, purified as described in Methods, were analyzed for listed genes using qPCR and specific primers; the amplified products were separated in agarose gel and representative blots from one representative individual are shown. (TIF) [file pone.0044915.s001.tif]

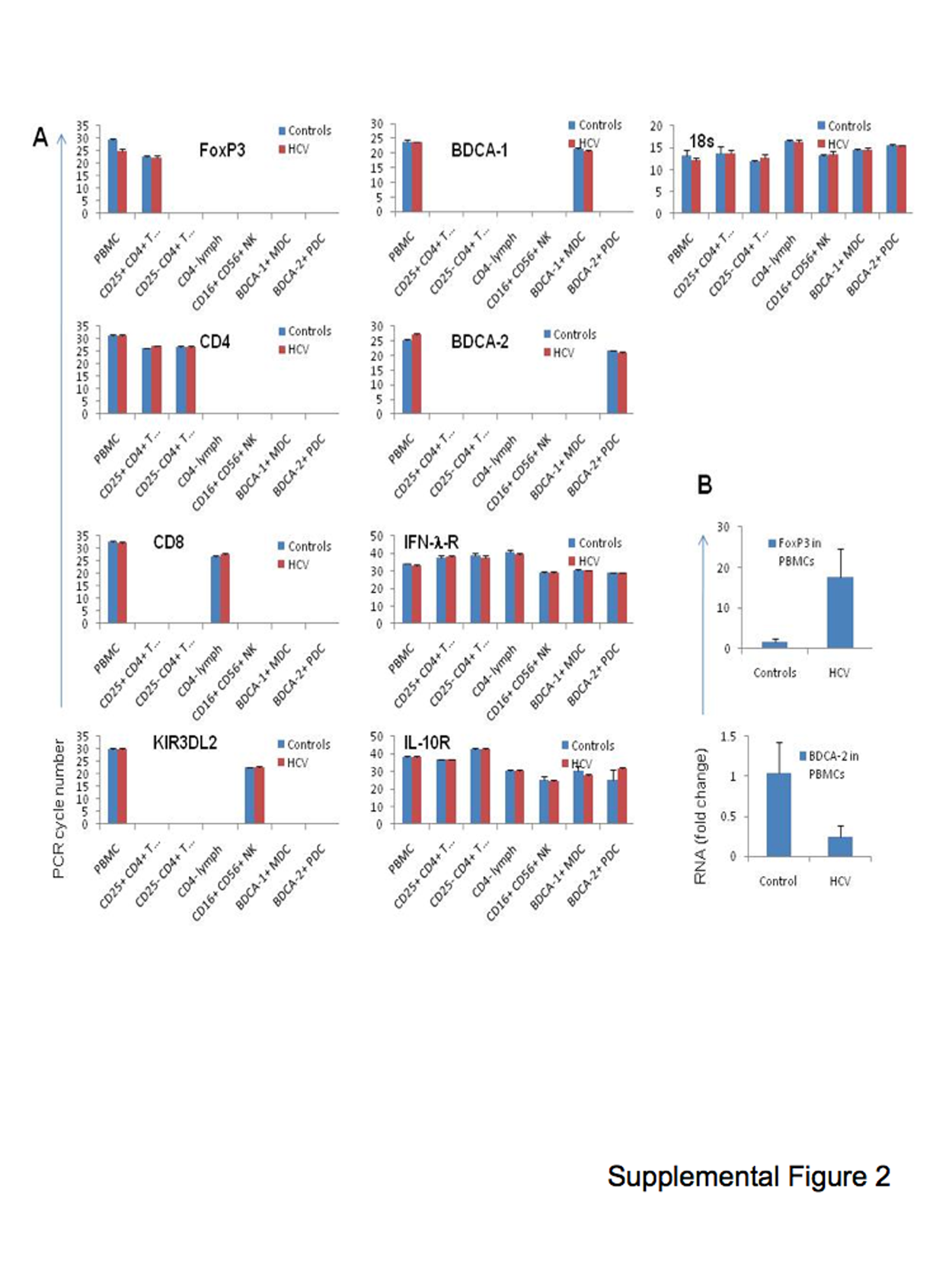

Supplement: Figure S2 — cHCV patients have increased levels of FoxP3 and reduced BDCA-2 in PBMCs compared to controls. Cell populations were isolated based on specific markers, as described in methods. Equal amounts of total cellular RNA was transcribed to DNA and analyzed for expression of specific markers by qPCR using SYBRgreen and specific primers. (A) PCR cycles, adjusted to housekeeping 18S control, are shown as mean ±SD. (B) The mean RNA levels of specific markers (FoxP3-top, BDCA-2-bottom) in PBMCs of controls were considered as equal to 1 and the fold change in HCV patient groups compared to controls was calculated (shown as mean±SD fold). * indicates p<0.05. (TIF) [file pone.0044915.s002.tif]

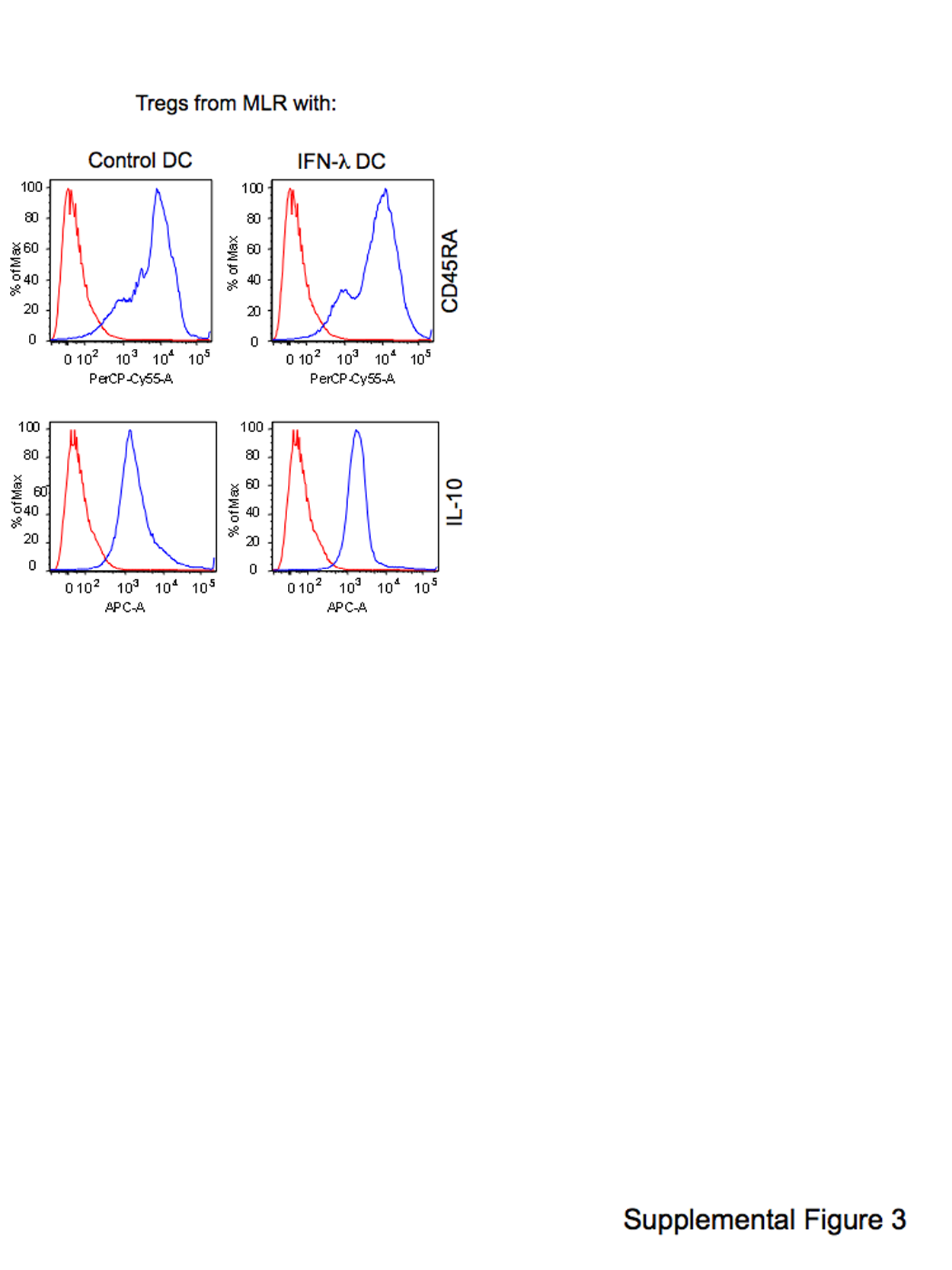

Supplement: Figure S3 — Tregs expanded by IFN- λ-exposed DCs have a regulatory phenotype. T cells were co-cultured with either control or IFN- λ-exposed DCs for 10 days, after which they were stimulated with PMA+Ionomycin for 4 hrs in the presence of GolgiStop. Cells were permeabilized, stained with specific antibodies, fixed, and analyzed by flow cytometry. Tregs were identified as CD4+CD25+FoxP3+ and analyzed for expression of CD45RA and IL-10 as indicated. Representative histograms of n = 5 are shown. (TIF) [file pone.0044915.s003.tif]
