# Supplementary material for: Type III Interferons, IL-28 and IL-29, Are Increased in Chronic HCV Infection and Induce Myeloid Dendritic Cell-Mediated FoxP3+ Regulatory T Cells
Source: PLoS One. 2012 Oct 10;7(10):e44915. doi: 10.1371/journal.pone.0044915 (PMC3468613; doi:10.1371/journal.pone.0044915)
Supplement: Table S1 — Primers for PCR analyses. (DOC) [file pone.0044915.s004.doc]

**Supporting Table 1. Primers for PCR analyses.**

| **Gene** | **Forward Primer** | **Reverse Primer** |
| --- | --- | --- |
| FoxP3 | tcacctacgccacggtcat | cacaaagcacttgtgcag |
| CD4 | ctaagctccagatgggcaag | ctgagtggctctcatcacca |
| CD8 | gctgccaaggactggagtag | acgggtggagagaggttttt |
| CD25 | cctgggagtcagaaaagctg | tgcttctctttgcattgtgg |
| BDCA-2 | ccaccatgcttggctaattt | aagaattttgtggccaggtg |
| BDCA-1(CD1c) | ggccaggacatcatcctcta | tgggttgctgggttcttaac |
| KIR3DL1 | actgcctgctggaaagaaaa | ggaacagcacgtgggtaagt |
| IFN-λ-R | cagccagtccagatcactctcc | tcctcaatttctgattccctcg |
| IL-10R | ggctgaatttgcagatgagca | gaagaccgaggccatgagg |
| IL-28A | ctcaggttgcatgactggtgg | gaggcctctgtcaccttcaac |
| IL-29 | ggacgccttggaagagtcact | agaagcctcaggtcccaattc |
| 18S | gtaacccgttgaaccccatt | ccatccaatcggtagtagcg |
